# Could Neonatal Hypernatremia Dehydration Influence Hearing Status?

**Published:** 2014-01

**Authors:** Hassan Boskabadi, Farnaz Anvarifar, Navid Nourizadeh

**Affiliations:** 1 *Department of Pediatrics , Ghaem Hospital, Mashhad University of Medical Sciences, Mashhad, Iran.*; 2 *Department of Otorhinolaryngology, Ghaem Hospital, Mashhad University of Medical Sciences, Mashhad, Iran.*

**Keywords:** Auditory Brainstem Response, Hearing loss, Hypernatremic dehydration, Newborn, Otoacustic emissions

## Abstract

**Introduction:**

Neonatal hypernatremia dehydration (NHD) is a dangerous condition in neonates, which is accompanied by acute complications (renal failure, cerebral edema, and cerebral hemorrhage) and chronic complications (developmental delay). Children begin learning language from birth, and hearing impairment interferes with this process. We assessed the hearing status of infants with hypernatremia dehydration.

**Materials and Methods:**

In a case-control study in 110 infants presenting at the Ghaem Hospital (Mashhad, Iran) between 2007 and 2011, we examined the incidence of hearing impairment in infants suffering from hypernatremia dehydration (serum sodium >150 mEq/L) in comparison with infants with normal sodium level (serum sodium ≤150 mEq/L).

**Results:**

Three of 110 cases examined in the study group showed a transient hearing impairment. A mean serum sodium level of 173mg/dl was reported among hearing-impaired infants.

**Conclusion:**

Transient hearing impairment was higher in infants with hypernatremia; although this difference was not significant (P>0.05). Hearing impairment was observed in cases of severe hypernatremia.

## Introduction

Neonatal hypernatremia dehydration (NHD) is a potentially dangerous condition often caused by inadequate nutrition during the first week of life (1). Numerous problems such as the mother’s lack of experience, the use of supplements, and the mother’s inadequate education may lead to excessive weight loss in infants in the first few days (2). The infant will respond through sodium retention to preserve water and reduce urination; which, if not treated, may result in NHD. Hypernatremia may be accompanied by such acute and fatal conditions as cerebral edema, seizure, cerebral hemorrhage, stroke, renal failure, and gangrene of the extremities (1–3). Few studies have been published on the long-term prognosis of this condition in neonates, and the existing studies have yielded conflicting results. Since the child starts to learn the language at birth, hearing loss (even unilaterally) can disturb this process, leading to speech impairment (4). Therefore, early diagnosis and early rehabilitation measures are of great significance. In a case-control study, we examined the hearing of NHD infants and studied the potential effect of NHD on hearing impairment.

## Materials and Methods

This case-control study was conducted on 1–29-day old infants presenting to the Ghaem Hospital and those hospitalized at the neonatal intensive care unit (NICU) and Emergency Ward. The case group consisted of 50 neonates hospitalized at the NICU and infants with sodium levels higher than 150 mg/dl, and the control group consisted of 65 infants brought to the hospital for routine check-up or physiological jaundice, with sodium levels lower than 150 mEq/L, and bilirubin levels lower than 15 mg/dl. Infants with hearing impairment of any origin and those with its risk factors were excluded from the study. On enrollment, a checklist consisting of the following items was completed: demographics, birth weight, current weight, pregnancy age, the Apgar score, duration of hospitalization, presence/absence of anomalies, symptom at hospital admission, breastfeeding status, feeding types (using or not using supplements), frequencies of urination and defecation, breast condition and problems, mother’s age, mother’s medical history and delivery method. Laboratory data (levels of sodium, potassium, urea, creatinine, and blood sugar) and para clinical tests (brain computed tomography [CT] scan and renal ultrasonography) were reported.

Hearing status based on OAE and ABR tests Infants in both groups underwent an otoacoustic emissions (OAE) test by an audiologist in addition to an ear examination. Infants with abnormal OAE underwent a further OAE test at the age of one month; and if the abnormality persisted, they underwent an auditory brainstem response (ABR) test. In case of ABR abnormality, this test was repeated every three months. We then examined and compared hearing impairment incidence in NHD neonates and neonates with normal sodium levels. The data were analyzed with SPSS 16, and P<0.05 was considered a significant value.

## Results

In this study, questionnaires were completed for 120 neonates, two of whom were excluded for a family history of hearing impairment, one for otitis media, and seven because their parents did not return for follow-up; hence, 110 neonates were studied.

In the case group, the mean duration of NHD was nine days, and the mean weight loss was 11.7%. The peak incidence in the case group was at the sixth day after birth. The chief cause of admission included poor feeding and fidgetiness, followed by lethargy, hyperthermia, and jaundice. The on-admission complaints and findings in the case group after weight loss (60%) were poor feeding 44%, hyperthermia 38%, fidgetiness 34%, lethargy 32%, mucosal dryness 16%, seizure 14%, jaundice 14%, apnea 4%, decline in consciousness 2%, and cyanosis 2%.

 In our study, most mothers were between 23 and 26 years of age, and the highest incidence was found in the first parity (66%). In our study, there was no significant relationship between the incidence of hypernatremia and the time of first breastfeeding, the mother’s age, pregnancy complications or duration of breastfeeding (P>0.05), but there was a significant relationship between the incidence of hypernatremia and the use of supplements in addition to breastfeeding, frequency of feeding and frequency of urination (P<0.05). The mean feeding frequency was 7 and 12 occasions in 24 hours in the case group and control group, respectively. Cerebral problems occurred in 8% of the neonates, three of whom had cerebral edema and one, a cerebral hemor- rhage.

In the case group, three neonates had an abnormal OAE at the onset of hypernatremia, followed by a normal OAE at the age of one month. Thus, the incidence of hearing impairment in this study “at the onset of hypernatremia” was 0% in the control group and 6% in the case group, and the statistical difference was not significant (P>0.05). The mean plasma sodium level in neonates with hearing impairment was higher than among controls (mean 173.667, SD 12.05 mEq).

Hypernatremic neonates with hearing impairment had a lower serum potassium and a higher creatinine compared with neonates with normal hearing (P<0.05). The former also had a lower mean blood sugar and higher age, although these differences were not statistically significant (Table. 1).

**Table 1:** Relationship between blood component in hypernatremia and impaired hearing

Serum level	Hearing loss	Normal hearing	P-value
Sodium	*173.66±12.05	165.17±12.96	0.275
Sugar	58.66±26/85	127.57±144.30	0.173
Creatinine	6.00±3.29	2.34±2.27	0.034
Urea	307.00±87.61	143.87±121.97	0.029
Potassium	3.73±1.95	5.68±1.36	0.03

* The values ​​are mean±SD

## Discussion

Hearing impairment is an asymptomatic disability in children, as speech development in children is delayed secondary to hearing impairment. Any delay in the diagnosis of hearing impairment can lead to adverse and irreversible consequences for the child (3,5). Unfortunately, since even relatively obvious hearing impairments are not usually diagnosed until the age of 2–3 years, this will not only disturb speech development but also have detrimental effects on the development of children’s social, communication, and educational skills (7). Hearing impairment of any degree adversely affects the emotional and educational development of children. Even mild hearing impairment exposes children to learning and speech problems (3,8). Since children start to learn to speak at birth, hearing impair- rment, even unilateral- lly, can disturb this process, and the child will develop speech impairment. Thus, early diagnosis and early rehabilitation measures are of great significance.

In our study, the age of the children in the control groups was three days younger than that in the case group (6±3.6 as opposed to 9±6.6 days), which shows that NHD infants are admitted towards the end of their first week. Likewise, in a study by Gomes (7), the mean age of the infants in the case group was four days, and in Jarcan’s study (8) the mean age of the infants with hypernatremia was six days. It therefore seems that routine neonatal visits in the middle of the first week (days 3 to 5) can help diagnose poor feeding and reduce the incidence of NHD.

In our study, there was no significant relationship between the incidence of hypernatremia and the time of first breastfeeding, mother’s age, pregnancy complications, or breastfeeding duration (P>0.05), but there was a significant relationship with number of feeding times per day (P=0.00). In others studies, there was also no relationship between the incidence of hypernatremia and the time of first breastfeeding (9,11). In some studies, the time to first breastfeeding was longer in the hypernatremic group than in the non-hypernatremic group (1,12). In our study, there was a significant relationship between the incidence of hypernatremia and the use of supplements in addition to breastfeeding. In the case group, 60% had used supple- ments in addition to breastfeeding compared with 21.7% in the control group (2,12).

In this study, the most frequent neonatal problem in the case group was weight loss; which was severe (over 10%) in 60% of the neonates. As for other symptoms, the most frequent on-admission complaints and findings in the case group after weight loss (60%) in view of accompanying findings in the case group were poor feeding (44%), hyperthermia (38%), fidgetiness (34%), lethargy (32%), mucosal dryness (16%), seizure (14%), jaundice (14%), apnea (4%), reduced consciousness (2%), and cyanosis(2%).

In this study, the range of sodium levels in the case group was 150–200 mEq/L and in the control group, 131–149 mEq/L. The mean sodium level in the case group was 165.7±12.9 mEq/L and in the control group, 141.18±4.71 mEq/L. The range of sodium levels in our study was similar to that in others study (1,12,13).

In our study, 8% of the infants developed cerebral complication (three infants [6%] cerebral edema, and one infant [2%] cerebral hemorrhage), which is similar to a study by Morris-Jones et al, in which 12 infants (37%) had abnormal neurological examinations and 9% suffered from major neurological conditions (14). In the study by Unal et al (15), several consequences of hypernatremic dehydration were described, including cerebral edema (5.2%), cerebral hemorrhage (3.6%), and cavernous sinus thrombosis (1.2%). Ten infants (5.9%) had seizures during the first 24 hours of fluid therapy, and two infants (1.2%) expired. This study has concluded that NHD can harm the central nervous system. Therefore, infants’ follow-up is important and pediatricians should be acutely aware of the possibility of hypernatremia, especially among neonates with pathologi- cal weight losses (15). Regarding the hearing status of the infants, it should be noted that clear hearing loss occurs with an incidence of 1/1000 at birth, and more often than not is unaccompanied by another diagnosable problem (3,7). 

In this study, none of the infants had abnormalities in the OAE in the control group. In the case group, three infants had abnormal OAE at the onset of hypernat- remia, but their subsequent OAE at the age of one month was normal. Thus, the incidence of hearing impairment in this study “at the onset of hypernatremia” was zero percent in the control group and 6% in the case group, a statistically insignificant difference (P=0.09). Therefore, there is no statistically significant relationship between NHD and hearing impairment. Noneth- eless, this may be an artifact of the small size of the sample in this study. It should be noted that the incidence of hearing impairment in NHD infants in our study was 60 times greater than that reported worldwide, which seems like a significant difference.

Of the three hearing-impaired infants, two had severe hypernatremia and one mild hypernatremia, and the mean plasma sodium level in the hearing-impaired infants was greater than in infants with normal hearing (mean 173.667 mEq/L, Vs 151.37 mEq/L, P=0.002). Therefore, it can be concluded that hearing impairment occurs with high levels of sodium. Likewise, in the study by Pasajlu and the study by Morris-Jones et al, hypernatremic complications occurred with higher levels of sodium. These complica- tions were also more frequent with higher levels of blood urea (14). Also in a further study, severe developmental delays occurred with more severe hypernatremia (1). 

It should be noted that changes in the endolymph and perilymph in the middle ear are not independent of the body’s hemodynamics, as can be seen in Meniere's disease. In this disease, endolymphatic hydrops causes dizziness, tinnitus, and fluctuated sensorineural hearing loss. Certain studies have recognized the contribution of the sudden combination of the perilymph and endolymph due to the rupture of the membranous labyrinth to this disease, which leads to physical and chemical changes in the vestibulocochlear system (16). Diuretics and limiting of dietary sodium are used in the treatment of this disease (17). It can therefore be suggested that hypernatremic dehydration can cause hearing impairment in infants by disturbing the balance between the endolymph and perilymph and affecting the pressure in the middle ear. Nonetheless, the confirmation or rejection of this hypothesis requires further and more extensive studies.

## Conclusion

Overall, it can be concluded that hypernatremic dehydration, especially in severe cases, can lead to transient abnormalities in audiometric tests, although in our study there was no significant statistical difference between the case and control groups in terms of the incidence of hearing impairment, which may be due to the small size of the sample in this study.
